# Gastroesophageal reflux disease: recent innovations in endoscopic assessment and treatment

**DOI:** 10.1093/gastro/goab029

**Published:** 2021-10-18

**Authors:** Sheng Chen, Feng Du, Changqing Zhong, Caifang Liu, Xiaoying Wang, Yan Chen, Gang Wang, Xiaopei Gao, Lu Zhang, Lianyong Li, Wei Wu

**Affiliations:** 1 Department of Gastroenterology, PLA Strategic Support Force Characteristic Medical Center, Beijing, P. R. China; 2 State Environmental Protection Key Laboratory of Environmental Sense Organ Stress and Health, PLA Strategic Support Force Characteristic Medical Center, Beijing, P. R. China; 3 Department of Internal Medicine, The Hospital of the People's Liberation Army 63650 Corps, Malan, Xinjiang, P. R. China; 4 Department of Pediatrics, The First Affiliated Hospital of Xi'an Medical University, Xi’an, Shaanxi, P. R. China; 5 Department of Otorhinolaryngology Head and Neck Surgery, PLA Strategic Support Force Characteristic Medical Center, Beijing, P. R. China

**Keywords:** gastroesophageal reflux disease, endoscopy, biopsy, treatment

## Abstract

Innovations in endoscopy have brought about some impressive improvements in diagnosing and treating gastroesophageal reflux disease (GERD). GERD, as one of the most prevalent gastrointestinal disorders in the world, has always been on the cutting edge of endoscopic interventions. A primary diagnosis of GERD is based on symptoms and an initial trial of proton-pump inhibitor (PPI) therapy, which is devoid of adequately instructive value for therapeutic strategies. Endoscopy and optional biopsies can be used to directly observe and determine the abnormal structural and pathophysiological damage in the esophagus. The emergence of minimally invasive endoscopic therapy fills the gap between patients who are reluctant or insensitive to PPIs and candidates who are not indicated for surgical anti-reflux fundoplication. In this review, we discuss the utility of endoscopy and biopsy in patients with persistent GERD-related manifestations after proper medical anti-reflux treatment. Moreover, we portray a landscape of four current endoscopic GERD therapies and clarify the merits and disadvantages of each technique. Future research needs to concentrate on stratifying GERD patients based on personal conditions and elucidating the primary pathophysiology of GERD.

## Introduction

The prevalence of gastroesophageal reflux disease (GERD) in diverse regions of the world varies widely, from 2.5% to 51.2%. In particular, people aged ≥50 years, smokers, non-steroidal anti-inflammatory drug users, and obese people have a significantly increased incidence of GERD [1]. The diagnosis of GERD depends on esophageal mucosal damage and reflux-related symptoms, the most typical of which are frequent troublesome heartburn and/or acid regurgitation [2]. Empirical proton-pump inhibitor (PPI) treatment also contributes to diagnosing GERD [3]. Although anti-secretory-based therapeutic diagnosis is appropriate for the initial step, when GERD is accompanied by alert symptoms or complications that are suggestive of a complicated condition, endoscopy and biopsy should be performed as soon as possible [4].

The treatment for GERD is mainly as follows: (i) lifestyle changes; (ii) medication including PPIs, H2 receptor antagonists, reflux-reducing agents, and adjunct medication; (iii) invasive management including anti-reflux surgery (ARS), bariatric surgery, magnetic sphincter augmentation, and endoscopic therapy [5]. Although PPIs have absolute advantages in drug treatment, a large number of studies have indicated that long-term usage of PPIs can give rise to bacterial gastroenteritis, bone fractures, chronic kidney disease, etc., which has curbed patients’ enthusiasm for taking PPIs [6, 7]. Moreover, some patients with refractory GERD have only a partial response or a lack of any clinical response even after taking the maximum dosage of PPIs (twice the routine dosage) [8].

Given these adverse effects of PPIs, laparoscopic Nissen fundoplication (LNF) or Toupet fundoplication (LTF), known as the classic criterion for ARS, has been applied clinically for nearly two decades, and each technique has continuously developed with various amounts of data supporting its role in improving the patient’s condition [3]. ARS, from which patients with severely disrupted esophagogastric junction (EGJ) structures (hiatus hernia >2 cm) would benefit, can control reflux attacks in 90% of patients for >10 years. However, currently, only approximately 0.05% of GERD patients will eventually receive ARS [9].

**Figure 1.**
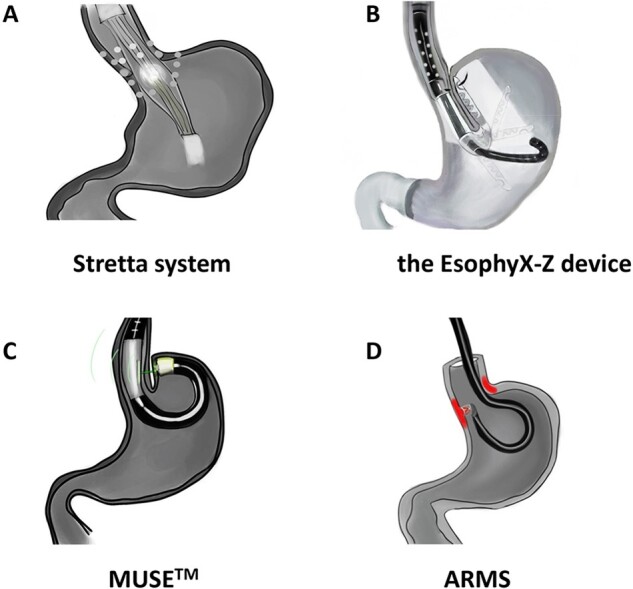
Schematic diagram of the four current endoscopic treatments in clinical practice. (A) The Stretta system: a flexible catheter to send a four-channel radio-frequency transmitter with titanium electrodes to the site which is 1 cm above the Z line and deliver the radio-frequency energy above and below the Z line. (B) The EsophyX-Z device: creation of an esophagogastric fundoplication proximal to the Z line. (C) MUSE^TM^: the tissue is clamped and sutured with the assistance of the ultrasound probe. (D) ARMS: 3-cm crescent-shaped mucosa cut in length is created (red) above and below Z line to remodel anti-reflux barrier with post-operative scar stenosis.

An increasing number of GERD patients are apt to resort to physicians and seek endoscopic assistance. At present, four kinds of endoscopic therapies are in clinical use, including radio-frequency ablation (RFA), transoral incisionless fundoplication (TIF), medigus ultrasonic surgical endostapler (MUSE^TM^), and anti-reflux mucosectomy (ARMS) (Figure 1). Each technique, to varying degrees, is effective. It has become clear that patients with GERD need individualized precision anti-reflux therapy according to their disease scenario and pathological characteristics to obtain the best and safest therapeutic benefit.

### Endoscopic evaluation and biopsies for GERD

For most patients, the diagnosis of GERD is based on empirical canonical symptoms and effective anti-secretory treatment [2, 3]. However, it is sensible and necessary for clinicians to conduct an endoscopic examination as soon as possible to make an accurate diagnosis or detect other diseases requiring alternative therapy if encountering the following conditions: (i) patient symptoms do not improve, or even worsen, after appropriate treatment; (ii) other symptoms occur (dysphagia, weight loss, hematemesis, choking, cough, hoarseness, asthma, laryngitis, chronic sore throat, or dental erosions); (iii) complications are suspected (such as hiatus hernia and Barrett’s esophagus [BE]); (iv) the necessity for placing pH-metry or pH-impedance; (v) requirement for laparoscopic or endoscopic anti-reflux therapy. If erythema, severe esophagitis, strictures, or BE are detected during the endoscopic examination, the diagnostic specificity of GERD can reach 95% [10]. In contrast, if the patient has only the typical symptoms of GERD, the sensitivity of the endoscopy examination is very low. Therefore, routine endoscopy is not recommended in those cases, since this kind of patient rarely has BE and erosions or just suffers from grade A esophagitis, which can also be found in 5%–7.5% of healthy people or controls without clinical symptoms.

Narrow banding imaging (NBI) can take advantage of real-time filtering of white light into narrow bands of green and blue light to observe irregular mucosal and vascular phenotypes to determine early proliferative damage [11], especially for patients with non-erosive gastroesophageal reflux disease (NERD). Mucosal damage in NERD is frequently difficult to probe under endoscopy with white light. The detection rate of abnormal blood vessels, non-round pit patterns, or epithelial micro injuries can be significantly improved under endoscopy equipped with NBI [12]. Furthermore, the dynamic ‘flap-valve’ structure, which belongs to the anti-reflux barrier, has some values in predicting GERD and it can be assessed through endoscopic retroflexion [13]. The Hill classification containing four degrees is used to evaluate the function and integrity of the flap valve [14]. Some research has pointed out the close relationship between the flap valve and reflux breakout [15, 16].

The role of biopsy in diagnosing GERD is still controversial and depends on the patients’ condition. Clinicians who have a negative attitude toward biopsy believe that although biopsy helps to identify eosinophilic esophagitis (EoE) in refractory GERD, the model put forward by Markov considers that only in the condition in which the prevalence of EoE is >8% is esophageal-biopsy cost-effective [17]. However, a high eosinophil count can also be found in some GERD patients or patients with PPI-responsive esophageal eosinophilia after a routine biopsy in the lower esophagus [18]. Notably, a biopsy is time-consuming and requires dedicated gastroesophageal pathologists. Therefore, routine biopsy is not recommended for patients with simple GERD manifestations or a normal appearance under endoscopy [19]. However, clinicians who support biopsy think that not only should samples of certain visible pathological changes such as ulceration, inflammation, lesions, and strictures be taken for further histopathological examination, but biopsy also has some values in distinguishing NERD from certain functional diseases with high sensitivity for reflux or functional heartburn, since some distinctive pathological changes can be observed in NERD mucosal histopathological samples [20].

Sometimes, improper sample collection affects the sensitivity of biopsy to diagnose GERD. Therefore, some studies have pointed out that the positive rate of the 3 o’clock position of the distal esophagus is the highest in patients with GERD who have obvious mucosal damage as well as in NERD patients [21]. A total esophageal epithelial thickness of 0.5 and 2 cm above the Z line >430 μm is a robust predictor of the presence of GERD [22, 23].

### Endoscopic anti-reflux management

Although ARS was once the most commonly applied treatment for refractory GERD, adverse reactions such as dysphagia, bloating, and increased flatus after surgery have led people to select endoscopic alternatives [24]. In the two decades after the development of endoscopic therapy, many types of devices, such as foreign-material filling transplants represented by Enteryx [25], EndoCinch that simulates the effects of surgical fundoplication [26], and Endoscopic Plicator systems, have been discarded due to a lack of long-term efficacy or safety [27]. At present, the Stretta system representing RFA and the EsophyX 2.0 device representing TIF are still widely applied in clinical practice. Stretta was approved by the US Food and Drug Administration in 2000 and has the most reliable long-term data. Curon Medical, Inc., which initially produced Stretta, went bankrupt in 2006. In 2008, Mederi Therapeutics, Inc. was authorized to restore Stretta to market again. Most studies involving TIF that confirmed its efficacy and safety have short-term follow-ups. Tables 1 and 2 summarize the study design, some subjective or objective parameters, and the reported complications of several major trials evaluating Stretta and TIF. MUSE^TM^ and ARMS are recently introduced techniques that have only been evaluated in small-scale observational studies but no randomized–controlled trials (RCTs).

**Table 1.** Brief profile of important studies evaluating Stretta

First author, year	Design	Patients	Symptom improvement	LESP (mmHg)	Acid exposure	Off PPIs	Complications
Reymunde [28], 2007	Prospective study F/U: 4 years	83 patients with persistent GERD symptoms	GERD-HRQL score: 2.4 (baseline) vs 4.6 (36 months) vs 4.3 (48 months) GERD symptom score: 2.7 (baseline) vs 0.3 (36 months) vs 0.6 (48 months)	NA	NA	0% (baseline) vs 86.4% (48 months)	NA
Arts [29], 2012	Double-blind randomized crossover study Stretta (*n* = 11) vs sham (*n* = 11) F/U: 3 months	22 patients diagnosed with GERD	Symptom scores: pre therapy (14.7) vs post therapy (8.3)	Stretta showed no change in LESP after 3 or 6 months GEJ compliance significantly decreased in Stretta	Both Stretta and sham did not significantly change acid exposure after 3 or 6 months	Both Stretta and sham did not decrease monthly PPI usage	NA
Lipka [30], 2015	Systematic review and meta-analysis 4 trials: 3 trials compared Stretta with sham + 1 trial compared Stretta with PPIs	153 patients diagnosed with GERD	HRQL scores: no improvement in Stretta compared with sham	No improvement in Stretta over sham	No significant benefit of Stretta over sham therapy	No advantage for stopping PPIs in Stretta	Stretta: aspiration pneumonia (1); gastroparesis (1)
Yan [31], 2015	Prospective study Stretta (*n* = 47) vs LTF (*n* = 51) F/U: 3 years	Patients diagnosed with GERD- related extraesophageal symptoms	Every symptom score including cough, sputum, wheezing improved in both groups without statistical significance except for globus hysterics	NA	NA	Stretta (61.7%) vs LTF (64.7%)	Stretta: fever (5); pharyngeal pain (9); retrosternal discomfort (14); diarrhea (4)
Fass [32], 2017	Systematic review and meta-analysis 28 studies (4 RCTs + 23 cohort studies + 1 registry) Mean F/U: 25.4 months	2,468 unique Stretta patients	HRQL score: reduced by 14.6; heartburn standardized score: reduced by 1.53	Increased by 1.73	Reduced by 3.01	2.9% (baseline) vs 65.5% (after Stretta)	Small erosions (9); mucosal lacerations (7); gastroparesis (3); bleeding esophageal ulcer (1); mediastinal inflammation (1); pleural effusion (1); pneumonia (1)
He [33], 2020	Prospective study Stretta (*n* = 28) vs PPI (n = 21) F/U: 6 months	Patients diagnosed with NERD	Symptom score: Stretta (6.3 ± 3.4) vs PPI (8.5 ± 4.1) Satisfaction rate: Stretta (89%) vs PPI (57%)	Stretta (14.2) vs PPI (10)	No significant improvement	Stretta (82%) vs PPI (52%)	Stretta: sore throat (2); mild fever (1); severe bloating and vomiting (1)

LESP, lower esophageal sphincter pressure; PPI, proton-pump inhibitor; GERD, gastroesophageal reflux disease; HRQL, Health Related Quality of Life; GEJ, gastroesophageal junction; NERD, non-erosive gastroesophageal reflux disease; NA, not available; LTF, laparoscopic Toupet fundoplication; F/U, follow-up.

**Table 2.** Brief profiles of important studies evaluating TIF

First author, year	Design	Patients	Symptom improvement	LESP (mmHg)	Acid exposure	Off PPIs	Complications
Bell [34], 2012	Prospective study F/U: 6 months	100 patients diagnosed with GERD	GERD-HRQL: 26 (baseline) vs 4 (6 months) RSI: 21 (baseline) vs 5 (6 months) GERSS: 29 (baseline) vs 4 (6 months)	NA	NA	8% (baseline) vs 80% (6 months)	NA
Witteman [35], 2015	RCT TIF 2.0 (*n* = 40) vs PPI (*n* = 20) F/U: 12 months	60 patients with chronic GERD	GERD-HRQL: TIF: 27.1 (baseline) vs 11.1 (6 months) vs 10.3 (12 months); PPI: 28.2 (baseline) vs 25.1 (6 months)	TIF: 15.3 (baseline) vs 17.8 (6 months) vs 17.6 (12 months) PPIs: 15.2 (baseline) vs 18.2 (6 months)	Total % time pH <4: TIF: 11.0 (baseline) vs 7.9 (6 months) vs 9.1 (12 months); PPIs: 11.3 (baseline) vs 6.0 (6 months) Total number of reflux episodes: TIF: 110.0 (baseline) vs 71.1 (6 months) vs 73.8 (12 months); PPIs: 109 (baseline) vs 101 (6 months)	TIF: 0% (baseline) vs 66% (6 months) vs 39% (12 months) PPIs: 0% (baseline) vs 0% (6 months)	TIF: pneumonia (3); severe epigastric pain (1); 1 death (cause uncertain) PPIs: NA
Richter [36], 2018	Systematic review and network meta-analysis of 7 RCTs	1,128 patients diagnosed with GERD	TIF is the best therapy for improving GERD-HRQL (SUCRA): TIF (0.96) vs LNF (0.66) vs sham (0.35) vs PPIs (0.042)	LNF holds the highest probability of increasing LESP (SUCRA): LNF (0.78) vs TIF (0.72) vs PPIs (0.01)	LNF is the most effective treatment for improvement in time% (pH <4) (SUCRA): LNF (0.99) vs PPI (0.64) vs TIF (0.32) vs sham (0.05)	NA	TIF: pneumonias (3); severe epigastric pain (1) LNF: infarctions (1) PPIs: infarctions (1); 1 death (pneumonia)
Chimukangara [37], 2019	Retrospective cohort study F/U: 8 years	57 patients diagnosed with GERD	GERD-HRQL score: 24 (baseline) vs 7 (12 months) vs 10 (8 years)	NA	NA	0% (baseline) vs 47% (12 months) vs 74% (8 years)	12 (21%) patients underwent LARS for recurrent GERD after TIF
Janu [38], 2019	Prospective cohort study Laparoscopic hiatal hernia repair + TIF 2.0 F/U: 12 months	99 patients with hiatal hernias between 2 and 5 cm	GERD-HRQL: 25.1 (baseline) vs 4.6 (6 months) vs 4.6 (12 months) GERSS: 25.0 (baseline) vs 2.0 (6 months) vs 1.0 (12 months) RSI: 26 (baseline) vs 15 (6 months) vs 16 (12 months)	NA	NA	4% (baseline) vs 70% (6 months) vs 74% (12 months)	NA

TIF, transoral incisionless fundoplication; LESP, lower esophageal sphincter pressure; PPI, proton-pump inhibitor; F/U, follow-up; GERD, gastroesophageal reflux disease; HRQL, Health Related Quality of Life; NA, not available; RSI, reflux symptom index; GERSS, gastroesophageal reflux symptom score; RCT, randomized–controlled trial; LARS, laparoscopic anti-reflux surgery; SUCRA, surface under the cumulative rank; LNF, laparoscopic Nissen fundoplication.

Compared with ARS, the four endoscopic treatments have more operational contraindications, including severe anatomical changes of the EGJ (>2 cm esophageal fissure hernia), severe esophageal lesions (grade C or D esophagitis, esophageal varices, esophageal stricture, BE, etc.), esophageal dysmotility, and obesity (body mass index >35) [39, 40]. Patients with the contraindications mentioned above should turn to other treatment options.

### RFA (the Stretta system)

This technique uses a flexible catheter to send a four-channel radio-frequency transmitter with titanium electrodes to 1 cm above the Z line. The electrodes are inserted into the esophageal muscle layer to deliver radio-frequency energy and the position is changed by rotating within 2 cm above and below the Z line for multipoint treatment [41]. The treatment principles of Stretta have not been clearly elucidated, but it can inhibit transient esophageal sphincter relaxation and reduce tissue compliance, thereby reducing reflux events and esophageal acid exposure. Stretta therapy has been proven safe. Common adverse complications include a small number of mucosal lacerations during treatment and post-operative short-term chest and pharyngeal pain, fever, and mucosal small erosions.

Two multicenter, prospective, and observational studies assessed 55 and 138 patients undergoing the Stretta procedure, respectively. The first study showed that the average GERD-Health Related Quality of Life (GERD-HRQL) score decreased to 15.2 post-operatively from 46.2 preoperatively after 771-day follow-up [42]. The second study with a 5-year follow-up evaluated the improvement in GERD-associated symptoms and found that the severity of heartburn, thoracalgia, and asthma was obviously relieved after the Stretta procedure, and 42.8% of patients completely ceased to take PPIs. Except for mild abdominal distention occurring in 12 patients post procedure in the second study, there were no severe complications observed in either study [43]. Another 8-year follow-up of 26 patients corroborated that heartburn and GERD-HRQL scores dropped at 4 and 8 years. Moreover, 20 patients ceased to take PPIs by the end of 8 years. However, the mean esophageal acid exposure time (AET) returned to baseline at 8 years after a dramatic decrease at 4 years [44]. The results of a 10-year follow-up with 217 medically refractory GERD patients undergoing Stretta were also released in 2014. Not only was the primary outcome (normalization of GERD-HRQL) attained in 72% of patients, but secondary outcomes (partially or completely off PPIs and an improvement in satisfaction score at 10 years) were also achieved in 64% and 54% of patients, respectively [45]. Overall, most prospective observational studies confirmed the positive and durable role of Stretta in improving symptoms and reducing PPI consumption. The encouraging results of observational studies paved the way for designing RCTs with higher quality. The first sham-controlled RCT regarding Stretta was published in 2003 and found that the principal outcomes (heartburn and GERD-HRQL scores) were satisfactory. Compared to sham treatment, patients with active treatment showed fewer heartburn attacks and obvious improvements in their GERD-HRQL scores at 6 and 12 months. Nevertheless, the secondary outcomes indicated no improvements in PPI use or AET [46]. The results of a trial published in 2010 were consistent with the first trial in reducing the GERD-HRQL score and the use of PPIs when compared with sham treatment. In addition, although no statistically significance of improvement including GERD-HRQL, mean 24-h pH, mean lower esophageal sphincter pressure (LESP), and PPI use were revealed between single- and double-Stretta therapy groups, numerical improvement in the double-Stretta group still indicated the promising efficacy for patients with dissatisfactory outcome of single Stretta [47]. Even in refractory patients who had previously received standard LNF, Stretta could still ameliorate the GERD-HRQL score and reduce medication use at 6 months or 10 years compared to the refractory LNF subset [48]. A recent systematic review and meta-analysis (including 4 RCTs, 23 cohort studies, and 1 registry) including 2,468 patients receiving Stretta demonstrated that Stretta could significantly reduce the occurrence of erosive esophagitis and the percentage of AET (pH <4) in a 24-h pH-screening period [32].

### TIF (the EsophyX-Z device)

TIF was approved by the US Food and Drug Administration in 2007 and some modifications have been made to make it more operational since TIF 1.0. The EsophyX-Z device, which represents TIF 2.0, was designed to mimic ARS to create 270° full-thickness esophagogastric fundoplication by using polypropylene H-transmural fasteners [49]. Many studies focusing on clinical outcomes, such as an improvement in symptoms, HRQL scores, and the withdrawal of PPIs, have been published. Recently, an 8-year cohort study assessed the long-term impact of TIF on the GERD-HRQL score and usage of PPIs. They found that 27% of patients had ceased to take daily PPI at longer follow-up and the median GERD-HRQL score decreased from 24 before the trial to 10 after 8 years. In addition, 78% of patients experienced relief of their symptoms, which demonstrated that TIF had a durable role in controlling the progression of GERD [37]. Another observational study in respect of reflux mechanisms also noted that TIF could decrease the number of postprandial transient lower esophageal sphincter relaxations (TLESRs) and total reflux episodes [50]. Not only subjective parameters, as mentioned above, but objective parameters also improved. The results of a study reporting 29 patients with hiatal hernia undergoing the TIF 2.0 procedure showed that objective parameters such as the mean pH score and acid pH exposure were also normalized [51].

A double-blind sham-controlled study of chronic PPI-dependent GERD patients performed in 2015 manifested that TIF 2.0 could offer significantly longer clinical remission than sham treatment. Moreover, outcomes of PPI consumption, AET, a reduction in scores, and the healing of reflux esophagitis also favored the TIF 2.0 procedure [52].

Some RCTs also confirmed the superiority of TIF over PPIs in controlling GERD evolution. A multicenter RCT in 2015 showed that 696 patients with troublesome regurgitation despite daily PPIs were randomized either to a group that received TIF and then placebo or to a group that received sham treatment and then omeprazole. The results at 6 months found elimination of regurgitation in 67% in the TIF group vs 45% in the PPIs group, and esophageal pH control also improved in TIF relative to sham [53]. A systematic review and meta-analysis including 32 studies (1,475 patients) was released in 2018. The results demonstrated some promising outcomes, such as GERD-HRQL, the gastroesophageal reflux symptom score, reflux symptom index, DeMeester scores, hernia reduction, and discontinuation of PPIs post TIF, which proved TIF to be safe and effective [54].

### Medigus ultrasonic surgical endostapler

The MUSE ^TM^ is an intraluminal fundoplication device integrated with an endoscope and consists of an endoscope, a camera, an ultrasound probe, and a suture device. During the procedure, the operator advances the MUSE^TM^ into the stomach through the marked EGJ and then the device is retroflexed to 270^°^. Once the proximal part of fundus is lifted against the shaft, the staples are fired from the cartridge and the stapling fundoplication is created [55, 56]. The outcomes of 66 patients receiving MUSE^TM^ after a follow-up of 6 months revealed an improvement in GERD-HRQL in 73% of off-PPI patients and a decrease in AET% with baseline PPI medication. However, at the beginning of the study, two patients experienced severe adverse events (pleural empyema and gastrointestinal bleeding) and required intervention [57]. For a longer follow-up of 4 years, 37 cases in the first study were analysed for the long-term safety and efficacy of MUSE^TM^. A total of 69.4% of patients were off PPIs and their GERD-HRQL score decreased significantly, which proved MUSE^TM^ to be relatively safe and efficacious in ameliorating GERD-associated symptoms as well as reducing PPI use [58].

### ARMS

ARMS is a relatively newfangled minimally invasive treatment for GERD that was inspired by the formation and contracture of mucosal scars after endoscopic mucosal resection or endoscopic submucosal dissection. Theoretically, the scar contracture caused by ARMS can narrow the EGJ, strengthen the flap valve, and reduce the occurrence of reflux [59]. In 2019, an Indian study reported the results of 62 GERD patients undergoing ARMS using cap-assisted endoscopic mucosal resection (ARMS-C) with a follow-up of 12 months. At 2 months, the mean DeMeester score returned to normal among 45 (72.5%) patients, and 43 (69.4%) patients had stopped using PPIs [60]. At 12 months, 38 (61.3%) patients expressed symptomatic relief and drug withdrawal through telephone interviews.

In the same year, a Korean study also confirmed the efficacy of ARMS-C among 33 GERD patients [61]. Six months after ARMS-C, 63% of patients discontinued PPIs and had significantly decreased GERD questionnaire scores and improved median DeMeester scores and AETs. Moreover, the median flap-valve grade and EGJ distensibility decreased. Some case reports of ARMS performed on five GERD patients in our hospital also concluded that ARMS is a feasible, effective, and safe treatment for GERD, but it requires more convincing assessments and conclusive evidence [62, 63].

### Choices between endoscopic treatment and ARS

For the past almost 70 years, ARS including LNF or LTF has been proven to be safe, with fewer side effects and satisfactory durability. However, enthusiasm for ARS declined after the peak in 2009. In 2020, Ma *et al.* [64] evaluated the outcomes of patients who underwent LTF and RFA. After 12 months’ follow-up, reflux time and frequency showed no difference in two groups. However, after multivariate Cox proportional regression analysis, RFA can increase the esophageal pH and pressure without improving the risk of poor prognosis. In 2015, a study compared the Stretta procedure and LTF; two groups almost achieved similar PPI independence and GERD-related extraesophageal symptoms [31]. A systematic review and network meta-analysis published in 2018 studied the efficacy of LNF, TIF, and PPI in controlling GERD. The results showed that TIF had the highest probability of subjective symptom relief while LNF had the highest ability to improve physiologic parameters [36]. In summary, the studies of the comparison between the traditional ARS and endoscopic treatment did not obtain a consistent conclusion. In many aspects, ARS and endoscopic treatment break even. In our opinion, ARS can be taken into consideration if mucosal damage still exists after maximal medical therapy or the severely structural disruption of EGJ is found.

### Current problems and future perspectives

GERD is an extremely common condition with a heterogeneous symptom profile and a multifaceted pathogenic basis, especially in Western countries. The economic cost, which is mostly derived from diagnostic tests and PPI use, is incredibly burdensome [65]. There is no doubt that making an accurate diagnosis and exploiting economical therapeutic methods are the two most vital aspects of conquering GERD today and in the future. At present, to our knowledge, authoritative guidelines do not recommend routine endoscopy examination for patients with typical and uncomplicated GERD. However, if clinicians are suspicious of complicated GERD (alarm symptoms, non-response to PPI therapy for 2 months, and multiple risk factors for BE), the performance of an endoscopy examination is advised. Similarly, the guidelines also recommend against routine tissue collection if endoscopic assessment indicates uncomplicated GERD or it excludes diseases such as EoE or BE [4].

We must acknowledge that no single diagnostic approach is perfect, especially for complicated GERD. Coming up with comprehensive diagnostic approaches with novel metrics for assessing functional impairment of the gastroesophageal flap valve or esophageal clearance ability as well as any underlying pathophysiological damage or aberrant visceral sensitivity in the anti-reflux barrier is crucial for facilitating tailored therapy for GERD.

Originally, Stretta was invented with the expectation that tissue remodeling of the lower esophageal sphincter (LES) could lead to an increase in LESP and a decrease in TLESRs. Although Stretta is safe and relatively easy to conduct, we should keep several caveats in mind that the studies that have proven the effectiveness of Stretta were restricted to subjective indicators such as GERD-HRQL scores or symptoms. A systematic review and meta-analysis published in 2015 reported that, compared to sham conditions, some objective parameters, such as the percentage of AET (pH <4) over a 24-h pH-screening period, LESP, the ability to stop PPIs, and the GERD-HRQL, did not change significantly after Stretta. The author suggested more objective data from high-resolution manometry, impedance–pH testing, and LES compliance tests should be obtained to assess the effectiveness and risks of the Stretta procedure [30]. Some meta-analyses also noted that the efficacy of Stretta was equal to traditional LNF or LTF in controlling GERD-related severe asthmatic or other extraesophageal symptoms [31, 66]. Hence, some guidelines and reviews have negative opinions about the Stretta system [3, 5].

Likewise, some RCTs have also questioned the efficacy of TIF, especially regarding long-term outcomes. A 12-month follow-up trial in 2015 compared the effectiveness of TIF with PPIs. Although TIF could result in symptom improvement at 6 months, no significant normalization of AET or pH was observed, and 61% of TIF patients resumed taking PPIs at 12 months [35]. A systematic review and network meta-analysis including 1,128 patients calculated that LNF almost outperformed TIF in all physiologic parameters, such as LESP and AET, but not the GERD-HRQL score. Therefore, the author did not recommend TIF as a long-term alternative to LNF or PPIs [36]. It seems that different academic institutions have opposite attitudes towards TIF and LNF. The American College of Gastroenterology does not recommend TIF as a long-term substitute for LNF [3], yet the Society of American Gastrointestinal and Endoscopic Surgeons supported TIF clinically in its published guidelines [49].

It is likely that innovations such as MUSE^TM^ and ARMS will continue to emerge as effective and durable therapies for GERD. However, relevant clinical data concerning MUSE^TM^ and ARMS, especially RCTs, are still scarce. Confronting criticism, it is admitted that there remain many areas for exploration and refinement, since RFA or TIF are far from their optimal potential. For example, it is worth probing whether extending 270° fundoplication could create a more durable and effective result with TIF. Additionally, whether a combination of two kinds of invasive therapies can result in a double-overlapping effect and compensate for individual shortcomings remains unclear. A recent trial investigated the effect of a combination of MUSE^TM^ and TIF, and satisfactory results showed that TIF with MUSE^TM^ obviously improved the GERD-related symptoms; >90% of patients reduced or discontinued PPIs use [67].

Overall, endoscopic interventions as the bridge between medication and ARS are attractive options for refractory GERD patients. The overall success of this approach requires clinicians to select patients using reasonable criteria and to modify the present treatments aiming at the primary pathophysiology of GERD.

## Authors' Contributions

W.W. and L.L. provided the idea for the review and revised the manuscript. C.L., X.W., Y.C., G.W., X.G., and L.Z. performed the literature search. S.C., F.D., and C.Z. conducted the data analysis and drafted the manuscript. All authors read and approved the final manuscript.

## Funding

None.

## References

[1] Eusebi LH , Ratnakumaran R, Yuan Y et al Global prevalence of, and risk factors for, gastro-oesophageal reflux symptoms: a meta-analysis. Gut 2018;67:430–40. 28232473 10.1136/gutjnl-2016-313589

[2] Gyawali CP , Kahrilas PJ, Savarino E et al Modern diagnosis of GERD: the Lyon Consensus. Gut 2018;67:1351–62. 29437910 10.1136/gutjnl-2017-314722 PMC6031267

[3] Katz PO , Gerson LB, Vela MF. Guidelines for the diagnosis and management of gastroesophageal reflux disease. Am J Gastroenterol 2013;108:308–28. 23419381 10.1038/ajg.2012.444

[4] Muthusamy VR , Lightdale JR, Acosta RD et al; Committee ASoP. The role of endoscopy in the management of GERD. Gastrointest Endosc 2015;81:1305–10. 25863867 10.1016/j.gie.2015.02.021

[5] Gyawali CP , Fass R. Management of gastroesophageal reflux disease. Gastroenterology 2018;154:302–18. 28827081 10.1053/j.gastro.2017.07.049

[6] Vaezi MF , Yang YX, Howden CW. Complications of proton pump inhibitor therapy. Gastroenterology 2017;153:35–48. 28528705 10.1053/j.gastro.2017.04.047

[7] Kia L , Kahrilas PJ. Gastroenterology KPJNr and hepatology. Therapy: risks associated with chronic PPI use—signal or noise? Nat Rev Gastroenterol Hepatol 2016;13:253–4. 27006255 10.1038/nrgastro.2016.44

[8] Triadafilopoulos G. Endoscopic options for gastroesophageal reflux: where are we now and what does the future hold? Curr Gastroenterol Rep 2016;18:47. 27424219 10.1007/s11894-016-0521-1

[9] Khan F , Maradey-Romero C, Ganocy S et al Utilisation of surgical fundoplication for patients with gastro-oesophageal reflux disease in the USA has declined rapidly between 2009 and 2013. Aliment Pharmacol Ther 2016;43:1124–31. 27060607 10.1111/apt.13611

[10] Roman S , Gyawali CP, Savarino E et al; GERD consensus group. Ambulatory reflux monitoring for diagnosis of gastro-esophageal reflux disease: update of the Porto consensus and recommendations from an international consensus group. Neurogastroenterol Motil 2017;29:1. 10.1111/nmo.13067 28370768

[11] Sanghi V , Thota PN. Barrett's esophagus: novel strategies for screening and surveillance. Ther Adv Chronic Dis 2019;10:2040622319837851. 30937155 10.1177/2040622319837851 PMC6435879

[12] Parikh ND , Viana AV, Shah S et al Image-enhanced endoscopy is specific for the diagnosis of non-erosive gastroesophageal reflux disease. Scand J Gastroenterol 2018;53:260–4. 29368532 10.1080/00365521.2018.1430847 PMC6080852

[13] Xie C , Li Y, Zhang N et al Gastroesophageal flap valve reflected EGJ morphology and correlated to acid reflux. BMC Gastroenterol 2017;17:118. 29166876 10.1186/s12876-017-0693-7 PMC5700691

[14] Hansdotter I , Björ O, Andreasson A et al Hill classification is superior to the axial length of a hiatal hernia for assessment of the mechanical anti-reflux barrier at the gastroesophageal junction. Endosc Int Open 2016;4:E311–7. 27004249 10.1055/s-0042-101021 PMC4798936

[15] Wu W , Li L, Qu C et al Reflux finding score is associated with gastroesophageal flap valve status in patients with laryngopharyngeal reflux disease: a retrospective study. Sci Rep 2019;9:15744. 31673091 10.1038/s41598-019-52349-5 PMC6823359

[16] Kim GH , Kang DH, Song GA et al Gastroesophageal flap valve is associated with gastroesophageal and gastropharyngeal reflux. J Gastroenterol 2006;41:654–61. 16933002 10.1007/s00535-006-1819-9

[17] Miller SM , Goldstein JL, Gerson LB. Cost-effectiveness model of endoscopic biopsy for eosinophilic esophagitis in patients with refractory GERD. Am J Gastroenterol 2011;106:1439–45. 21448144 10.1038/ajg.2011.94

[18] Kia L , Hirano I. Distinguishing GERD from eosinophilic oesophagitis: concepts and controversies. Nat Rev Gastroenterol Hepatol 2015;12:379–86. 25986303 10.1038/nrgastro.2015.75 PMC4948861

[19] Takubo K , Honma N, Aryal G et al Is there a set of histologic changes that are invariably reflux associated? Arch Pathol Lab Med 2005;129:159–63. 15679411 10.5858/2005-129-159-ITASOH

[20] Aziz Q , Fass R, Gyawali CP et al Functional esophageal disorders. Gastroenterology 2016;150:1368–79. 10.1053/j.gastro.2016.02.012 27144625

[21] Edebo A , Vieth M, Tam W et al Circumferential and axial distribution of esophageal mucosal damage in reflux disease. Dis Esophagus 2007;20:232–8. 17509120 10.1111/j.1442-2050.2007.00678.x

[22] Vakil N , Vieth M, Wernersson B et al Diagnosis of gastro-oesophageal reflux disease is enhanced by adding oesophageal histology and excluding epigastric pain. Aliment Pharmacol Ther 2017;45:1350–7. 28318045 10.1111/apt.14028

[23] Vieth M , Mastracci L, Vakil N et al Epithelial thickness is a marker of gastroesophageal reflux disease. Clin Gastroenterol Hepatol 2016;14:1544–51. 27374007 10.1016/j.cgh.2016.06.018

[24] Yadlapati R , Hungness ES, Pandolfino JE. Complications of antireflux surgery. Am J Gastroenterol 2018;113:1137–47. 29899438 10.1038/s41395-018-0115-7 PMC6394217

[25] Johnson DA , Ganz R, Aisenberg J et al Endoscopic implantation of enteryx for treatment of GERD: 12-month results of a prospective, multicenter trial. Am J Gastroenterology 2003;98:1921–30. 10.1111/j.1572-0241.2003.08109.x 14499767

[26] Schwartz MP , Wellink H, Gooszen HG et al Endoscopic gastroplication for the treatment of gastro-oesophageal reflux disease: a randomised, sham-controlled trial. Gut 2007;56:20–8. 16763053 10.1136/gut.2006.096842 PMC1856666

[27] Rothstein R , Filipi C, Caca K et al Endoscopic full-thickness plication for the treatment of gastroesophageal reflux disease: a randomized, sham-controlled trial. Gastroenterology 2006;131:704–12. 16952539 10.1053/j.gastro.2006.07.004

[28] Reymunde A , Santiago N. Long-term results of radiofrequency energy delivery for the treatment of GERD: sustained improvements in symptoms, quality of life, and drug use at 4-year follow-up. Gastrointest Endosc 2007;65:361–6. 17321231 10.1016/j.gie.2006.06.036

[29] Arts J , Bisschops R, Blondeau K et al A double-blind sham-controlled study of the effect of radiofrequency energy on symptoms and distensibility of the gastro-esophageal junction in GERD. Am J Gastroenterol 2012;107:222–30. 22108449 10.1038/ajg.2011.395

[30] Lipka S , Kumar A, Richter JE. No evidence for efficacy of radiofrequency ablation for treatment of gastroesophageal reflux disease: a systematic review and meta-analysis. Clin Gastroenterol Hepatol 2015;13:1058–67. 25459556 10.1016/j.cgh.2014.10.013

[31] Yan C , Liang WT, Wang ZG et al Comparison of Stretta procedure and Toupet fundoplication for gastroesophageal reflux disease-related extra-esophageal symptoms. World J Gastroenterol 2015;21:12882–7. 26668513 10.3748/wjg.v21.i45.12882 PMC4671044

[32] Fass R , Cahn F, Scotti DJ et al Systematic review and meta-analysis of controlled and prospective cohort efficacy studies of endoscopic radiofrequency for treatment of gastroesophageal reflux disease. Surg Endosc 2017;31:4865–82. 28233093 10.1007/s00464-017-5431-2

[33] He S , Xu F, Xiong X et al Stretta procedure versus proton pump inhibitors for the treatment of nonerosive reflux disease: a 6-month follow-up. Medicine (Baltimore*)* 2020;99:e18610. 32011441 10.1097/MD.0000000000018610 PMC7220108

[34] Bell RC , Mavrelis PG, Barnes WE et al A prospective multicenter registry of patients with chronic gastroesophageal reflux disease receiving transoral incisionless fundoplication. J Am Coll Surg 2012;215:794–809. 22939637 10.1016/j.jamcollsurg.2012.07.014

[35] Witteman BP , Conchillo JM, Rinsma NF et al Randomized controlled trial of transoral incisionless fundoplication vs. proton pump inhibitors for treatment of gastroesophageal reflux disease. Am J Gastroenterol 2015;110:531–42. 25823768 10.1038/ajg.2015.28

[36] Richter JE , Kumar A, Lipka S et al Efficacy of laparoscopic Nissen fundoplication vs transoral incisionless fundoplication or proton pump inhibitors in patients with gastroesophageal reflux disease: a systematic review and network meta-analysis. Gastroenterology 2018;154:1298–308. 29305934 10.1053/j.gastro.2017.12.021

[37] Chimukangara M , Jalilvand AD, Melvin WS et al Long-term reported outcomes of transoral incisionless fundoplication: an 8-year cohort study. Surg Endosc 2019;33:1304–9. 30167944 10.1007/s00464-018-6403-x PMC6461469

[38] Janu P , Shughoury AB, Venkat K et al Laparoscopic hiatal hernia repair followed by transoral incisionless fundoplication with EsophyX device (HH + TIF): efficacy and safety in two community hospitals. Surg Innov 2019;26:675–86. 31431138 10.1177/1553350619869449 PMC6843624

[39] Testoni PA , Vailati C, Testoni S et al Transoral incisionless fundoplication (TIF 2.0) with EsophyX for gastroesophageal reflux disease: long-term results and findings affecting outcome. Surg Endosc 2012;26:1425–35. 22170317 10.1007/s00464-011-2050-1

[40] Witteman BPL , Strijkers R, de Vries E et al Transoral incisionless fundoplication for treatment of gastroesophageal reflux disease in clinical practice. Surg Endosc 2012;26:3307–15. 22648098 10.1007/s00464-012-2324-2 PMC3472060

[41] Richards WO , Scholz S, Khaitan L et al Initial experience with the stretta procedure for the treatment of gastroesophageal reflux disease. J Laparoendosc Adv Surg Tech A 2001;11:267–73. 11642661 10.1089/109264201317054546

[42] Viswanath Y , Maguire N, Obuobi RB et al Endoscopic day case antireflux radiofrequency (Stretta) therapy improves quality of life and reduce proton pump inhibitor (PPI) dependency in patients with gastro-oesophageal reflux disease: a prospective study from a UK tertiary centre. Frontline Gastroenterol 2019;10:113–9. 31205650 10.1136/flgastro-2018-101028 PMC6540282

[43] Liang WT , Wang ZG, Wang F et al Long-term outcomes of patients with refractory gastroesophageal reflux disease following a minimally invasive endoscopic procedure: a prospective observational study. BMC Gastroenterol 2014;14:178. 25304252 10.1186/1471-230X-14-178 PMC4287567

[44] Dughera L , Rotondano G, De Cento M et al Durability of Stretta radiofrequency treatment for GERD: results of an 8-year follow-up. Gastroenterol Res Pract 2014;2014:531907. 24959175 10.1155/2014/531907 PMC4052191

[45] Noar M , Squires P, Noar E et al Long-term maintenance effect of radiofrequency energy delivery for refractory GERD: a decade later. Surg Endosc 2014;28:2323–33. 24562599 10.1007/s00464-014-3461-6

[46] Corley DA , Katz P, Wo JM et al Improvement of gastroesophageal reflux symptoms after radiofrequency energy: a randomized, sham-controlled trial. Gastroenterology 2003;125:668–76. 12949712 10.1016/s0016-5085(03)01052-7

[47] Aziz AMA , El-Khayat HR, Sadek A et al A prospective randomized trial of sham, single-dose Stretta, and double-dose Stretta for the treatment of gastroesophageal reflux disease. Surg Endosc 2010;24:818–25. 19730952 10.1007/s00464-009-0671-4

[48] Noar M , Squires P, Khan S. Radiofrequency energy delivery to the lower esophageal sphincter improves gastroesophageal reflux patient-reported outcomes in failed laparoscopic Nissen fundoplication cohort. Surg Endosc 2017;31:2854–62. 28039654 10.1007/s00464-016-5296-9

[49] Smith CD. SAGES clinical spotlight review: endoluminal treatments for gastroesophageal reflux disease (GERD). Surg Endosc 2013;27:2655–7. 23689835 10.1007/s00464-013-3011-7

[50] Rinsma NF , Smeets FG, Bruls DW et al Effect of transoral incisionless fundoplication on reflux mechanisms. Surg Endosc 2014;28:941–9. 24149854 10.1007/s00464-013-3250-7

[51] Ihde GM 2nd , Pena C, Scitern C et al pH scores in hiatal repair with transoral incisionless fundoplication. JSLS 2019;23:e2018. 10.4293/JSLS.2018.00087 PMC6333564 30675094

[52] Håkansson B , Montgomery M, Cadiere GB et al Randomised clinical trial: transoral incisionless fundoplication vs. sham intervention to control chronic GERD. Aliment Pharmacol Ther 2015;42:1261–70. 26463242 10.1111/apt.13427

[53] Hunter JG , Kahrilas PJ, Bell RCW et al Efficacy of transoral fundoplication vs omeprazole for treatment of regurgitation in a randomized controlled trial. Gastroenterology 2015;148:324–33.e5. 25448925 10.1053/j.gastro.2014.10.009

[54] McCarty TR , Itidiare M, Njei B et al Efficacy of transoral incisionless fundoplication for refractory gastroesophageal reflux disease: a systematic review and meta-analysis. Endoscopy 2018;50:708–25. 29625507 10.1055/a-0576-6589

[55] Kauer WK , Roy-Shapira A, Watson D et al Preclinical trial of a modified gastroscope that performs a true anterior fundoplication for the endoluminal treatment of gastroesophageal reflux disease. Surg Endosc 2009;23:2728–31. 19357915 10.1007/s00464-009-0479-2

[56] Gweon TG , Matthes K. Prospective, randomized ex vivo trial to assess the ideal stapling site for endoscopic fundoplication with Medigus Ultrasonic Surgical Endostapler. Gastroenterol Res Pract 2016;2016:3161738. 27547219 10.1155/2016/3161738 PMC4983363

[57] Zacherl J , Roy-Shapira A, Bonavina L et al Endoscopic anterior fundoplication with the Medigus Ultrasonic Surgical Endostapler (MUSE™) for gastroesophageal reflux disease: 6-month results from a multi-center prospective trial. Surg Endosc 2015;29:220–9. 25135443 10.1007/s00464-014-3731-3 PMC4293474

[58] Kim HJ , Kwon C-I, Kessler WR et al Long-term follow-up results of endoscopic treatment of gastroesophageal reflux disease with the MUSE™ endoscopic stapling device. Surg Endosc 2016;30:3402–8. 26537905 10.1007/s00464-015-4622-y PMC4956714

[59] Ota K , Takeuchi T, Harada S et al A novel endoscopic submucosal dissection technique for proton pump inhibitor-refractory gastroesophageal reflux disease. Scand J Gastroenterol 2014;49:1409–13. 25384555 10.3109/00365521.2014.978815

[60] Patil G , Dalal A, Maydeo A. Feasibility and outcomes of anti-reflux mucosectomy (ARMS) for proton pump inhibitor dependent gastroesophageal reflux disease: first Indian study (with video). Dig Endosc 2020;32:745–52. 31834663 10.1111/den.13606

[61] Yoo IK , Ko WJ, Kim HS et al Anti-reflux mucosectomy using a cap-assisted endoscopic mucosal resection method for refractory gastroesophageal disease: a prospective feasibility study. Surg Endosc 2020;34:1124–31. 31139995 10.1007/s00464-019-06859-y

[62] Min M , Liu Y. A novel endoloop pretest to treat severe gastroesophageal reflux disease symptoms before anti-reflux mucosectomy. Endoscopy 2019;51:E193–4. 30978737 10.1055/a-0861-9849

[63] Monino L , Gonzalez J-M, Vitton V et al Anti-reflux mucosectomy with band ligation in the treatment of refractory gastroesophageal reflux disease. Endoscopy 2019;51:E215–6. 31039591 10.1055/a-0875-3479

[64] Ma L , Li T, Liu G et al Stretta radiofrequency treatment vs Toupet fundoplication for gastroesophageal reflux disease: a comparative study. BMC Gastroenterol 2020;20:162. 32460696 10.1186/s12876-020-01310-2 PMC7251847

[65] Shaheen NJ , Hansen RA, Morgan DR et al The burden of gastrointestinal and liver diseases, 2006. Am J Gastroenterol 2006;101:2128. 16848807 10.1111/j.1572-0241.2006.00723.x

[66] Hu Z , Wu J, Wang Z et al Outcome of Stretta radiofrequency and fundoplication for GERD-related severe asthmatic symptoms. Front Med 2015;9:437–43. 26566608 10.1007/s11684-015-0422-y

[67] Testoni PA , Testoni S, Mazzoleni G et al Transoral incisionless fundoplication with an ultrasonic surgical endostapler for the treatment of gastroesophageal reflux disease: 12-month outcomes. Endoscopy 2020;52:469–73. 32187630 10.1055/a-1124-3187

